# Evolving Insights on Metabolism, Autophagy, and Epigenetics in Liver Myofibroblasts

**DOI:** 10.3389/fphys.2016.00191

**Published:** 2016-06-01

**Authors:** Zeribe C. Nwosu, Hamed Alborzinia, Stefan Wölfl, Steven Dooley, Yan Liu

**Affiliations:** ^1^Molecular Hepatology Section, Department of Medicine II, Medical Faculty Mannheim, University of HeidelbergMannheim, Germany; ^2^Institute of Pharmacy and Molecular Biotechnology, University of HeidelbergHeidelberg, Germany

**Keywords:** liver myofibroblasts, metabolism, autophagy, epigenetics, fibrosis, hepatic stellate cells

## Abstract

Liver myofibroblasts (MFB) are crucial mediators of extracellular matrix (ECM) deposition in liver fibrosis. They arise mainly from hepatic stellate cells (HSCs) upon a process termed “activation.” To a lesser extent, and depending on the cause of liver damage, portal fibroblasts, mesothelial cells, and fibrocytes may also contribute to the MFB population. Targeting MFB to reduce liver fibrosis is currently an area of intense research. Unfortunately, a clog in the wheel of antifibrotic therapies is the fact that although MFB are known to mediate scar formation, and participate in liver inflammatory response, many of their molecular portraits are currently unknown. In this review, we discuss recent understanding of MFB in health and diseases, focusing specifically on three evolving research fields: metabolism, autophagy, and epigenetics. We have emphasized on therapeutic prospects where applicable and mentioned techniques for use in MFB studies. Subsequently, we highlighted uncharted territories in MFB research to help direct future efforts aimed at bridging gaps in current knowledge.

## Introduction

Long-term exposure of the liver to injurious xenobiotic insults is a major cause of liver fibrosis and its sequelae, notably cirrhosis, acute liver failure, and liver cancer. Fibrosis is characterized by the net accumulation of extracellular matrix (ECM) and scar formation. This process is driven by a heterogeneous population of liver myofibroblasts (MFB) that are recruited to and accumulate at the site of injury. Hepatic stellate cells (HSCs) are widely accepted as the major source of liver MFB. Studies have consistently shown that upon activation to MFB, HSCs play a crucial role in the development of liver fibrosis. The activation process is induced by various stimulatory factors, including transforming growth factor beta (TGF-β) and inflammatory cytokines (Dooley et al., 2003; Gabbiani, 2003; Mederacke et al., 2013; Seki and Schwabe, 2015). Besides HSCs, other cell types such as portal fibroblasts, bone marrow-derived fibrocytes and mesothelial cells may also contribute to liver MFB in response to chronic injury (Iwaisako et al., 2014; Xu et al., 2015).

Regardless of their origin, MFB are highly contractile, proliferative, and produce ECM components such as collagen types I, III, and fibronectin (Bataller and Brenner, 2005). MFB mediate reconstruction of connective tissues upon injury (Gabbiani, 2003; Swiderska-Syn et al., 2014). For example, following partial hepatectomy, MFB not only accumulate at the site of injury to initiate liver regeneration, but also activate liver progenitor cells, and subsequently induce proliferation of hepatocytes and cholangiocytes via hedgehog signaling pathway (Swiderska-Syn et al., 2014). It is therefore conceivable that extremely sophisticated mechanisms are responsible for the timely activation, recruitment, homing, and perpetuation of MFB functions at injured sites. There is also evidence that activated HSCs may undergo a coordinated reversion to quiescence once “their job” is done (Pellicoro et al., 2014).

In the last decade, new understanding of cellular metabolism arose especially with regards to cancer cells. This followed consistent *in vitro* and *in vivo* experimental proofs that tumor cells reprogram their metabolism to ensure continual survival (Vander Heiden et al., 2009; Hanahan and Weinberg, 2011). However, quite contrary to prevailing views, metabolic alterations or reprogramming are not exclusive to cancer cells. In fact, many other cell types, including dendritic cells, macrophages, T-cells, myeloid derived suppressor cells, cortical astrocytes, microglia, and skeletal muscle cells may also undergo metabolic changes under a variety of initiating factors (Bentaib et al., 2014; Gimeno-Bayón et al., 2014; Kelly and O'Neill, 2015; Maekawa et al., 2015; Pallett et al., 2015; Ryall et al., 2015; Shi et al., 2015; Xu et al., 2015). Hence, after years of focus on cell signaling, it is time to refocus efforts on how metabolic perturbations might influence the activity of MFB, including any therapeutic prospects it holds.

Closely linked to metabolism is autophagy (Galluzzi et al., 2014; Filomeni et al., 2015), and in many contexts, both processes have the same goal—energy generation. In autophagy, cells “eat up” their cellular components to produce sufficient energy to meet other immediate needs; however, autophagy could also be a cell death process (Elmore, 2007; Green and Levine, 2014). Such a dynamic system could be pivotal in MFB homeostasis. Metabolic alterations and autophagic responses may have epigenetic twists, e.g., via the transcriptional switch of critical gene networks (Hanley et al., 2010). Thus, epigenetic processes could enhance or suppress gene functions as the need arises during HSC-MFB transdifferentiation.

In this review, we have highlighted current knowledge on metabolism, autophagy and epigenetics in liver MFB. We also briefly mention recent technical advances that could help unravel new insights on the three topics in discourse. Finally, we offer perspectives to stimulate further questions on the role of metabolism, autophagy, and epigenetics in liver MFB.

## Metabolic alterations in liver myofibroblasts

**Figure 1:**
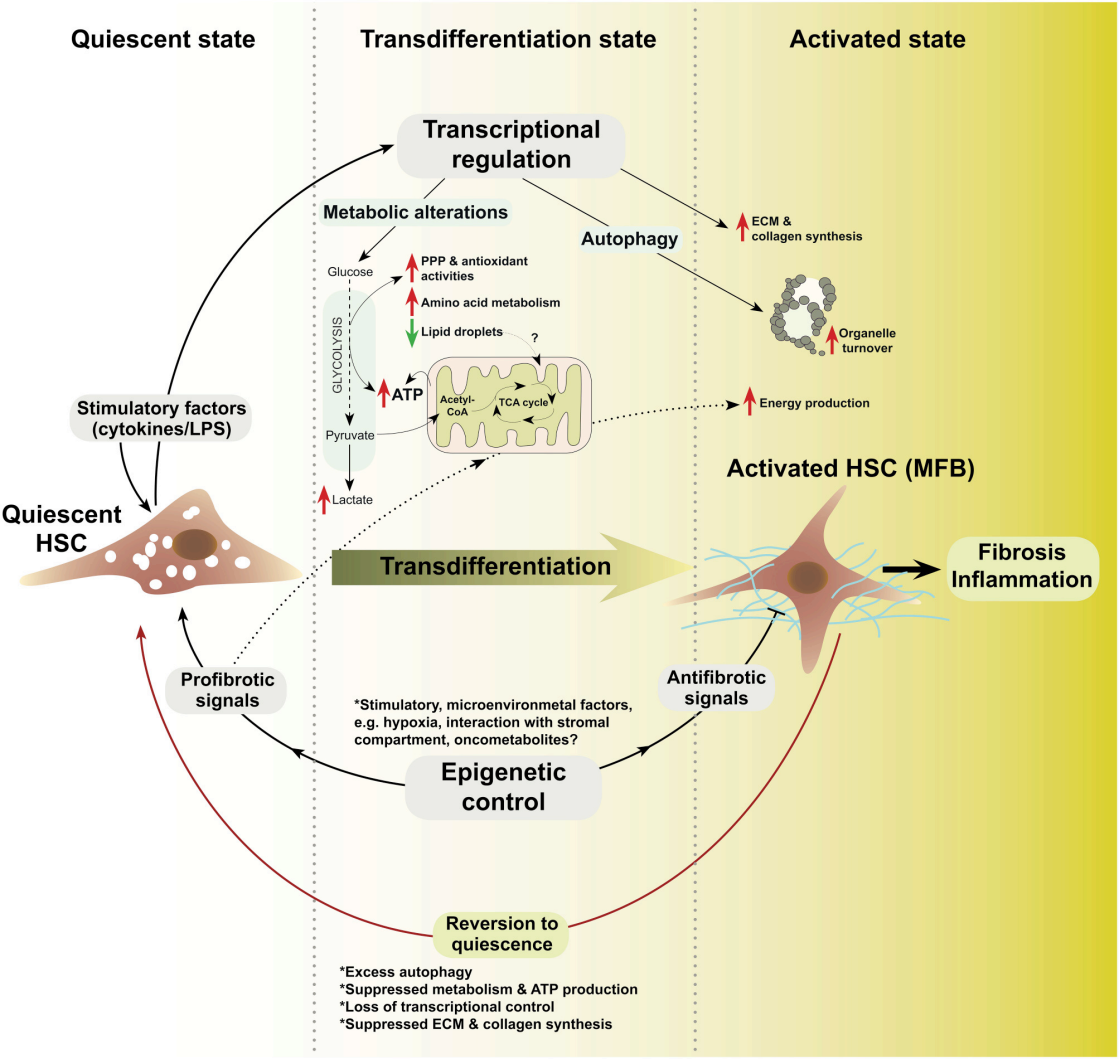
**A schematic model of metabolism, autophagy, and epigenetics in HSC-to-MFB transdifferentiation based on evolving research insights**. In this model, quiescent HSC exposed to various stimuli transdifferentiate to activated HSC (MFB) and loose lipid droplets (LDs). Prior to or during transdifferentiation, transcription-level alterations modulate the expression of relevant metabolic, autophagy, and epigenetic mediators (?). Epigenetic modifiers (e.g., HDACs, DNMTs, MECP2, etc.) may determine, which transcriptional networks are switched on or off. In the transdifferentiated state, increased glycolysis, pentose phosphate pathway (PPP), and antioxidant system as well as loss of LDs could synergize to sustain energy production (Hernández-Gea et al., 2012) and provide the metabolite pool for extracellular matrix (ECM) and collagen synthesis (?). In the activated state, MFB may rely on nutrients from accelerated *de novo* metabolism, microenvironment, or autophagic breakdown of organelles to sustain their function in fibrosis and inflammation, e.g., cytokine production. Microenvironmental factors may signal the end of healing by (a) activating antifibrotic epigenetic modifiers, (b) attenuating transcriptional activators of metabolism and autophagy, or (c) by inducing self-destructive autophagy in MFB (?). LPS, lipopolysaccharides; ?, unknown mechanisms.

There is a growing knowledge of metabolic alterations in various types of cells. Despite paucity of experimental evidences, it is plausible that metabolic alterations are critical in the transdifferentiation of HSCs to MFB. Key intermediary metabolic pathways previously implicated in malignant transformation and cell survival, may be intricately involved in the maintenance of membrane integrity, morphology, energy production, signaling among other functions in MFB. Thus, metabolism could control the balance between MFB and the reversal to quiescent HSCs (Figure 1).

### Glycolysis

The role of glycolysis in MFB origin or function is currently understudied. HSCs gain a glycolytic phenotype upon activation (Chen et al., 2012). Specifically, several glycolytic targets including GLUT1, HK2, PKM2, and lactate transporter MCT4 were simultaneously upregulated with alpha smooth muscle actin (α-SMA) during culture activation of HSCs and in animal liver fibrosis models (Chen et al., 2012). The glycolytic feature was mediated via Hedgehog (Hh) signaling and strongly correlated with expression of hypoxia inducible factor 1α (HIF1α), a known transcriptional regulator of glycolytic genes (Chen et al., 2012). Mechanistically, damaged hepatocytes release Hedgehog ligands, which activate HSCs via Hh signaling mediator Smoothened (SMO), and HIF1α induction. Deletion of SMO in quiescent HSCs suppressed basal mRNA expression of *Glut1, Hk2, Pkm2*, and HIF1α, while the opposite effect was observed with SMO agonist (SAG). Hence, the authors confirmed a direct link between MFB glycolytic activity and progression of liver fibrosis since inhibition of Hh signaling, HIF1α, glycolysis, or lactate accumulation all converted MFB to quiescent HSCs. In addition, the number of glycolytic stromal cells, as determined by *PKM2* expression, also correlated with the severity of fibrosis in diseased livers of animals and patients (Chen et al., 2012). Consistent with the above findings, Hh signaling inhibitors with potent antifibrotic effects (i.e., cyclopamine and curcumin) were recently shown to decrease intracellular levels of adenosine triphosphate (ATP), lactate, and the expression of glycolytic targets HK, PFK2, and Glut4 in HSCs (Lian et al., 2015). These evidences support the role of glycolysis in HSC activation and highlight the possibilities of targeting this metabolic pathway toward ameliorating fibrosis. Obviously, more studies are required to investigate the direct effect of modulating glycolytic targets in HSCs. Specifically, findings from MFB of other cellular origin could be tested in liver MFB. For example, glycolytic alterations are observed during MFB differentiation in the lung and prostate. Lung MFB at their early activation stage have increased and sustained expression of glycolytic enzymes PFK1, HK2, and notably PFKFB3. Inhibition of PFKFB3 with 3PO suppressed fibroblast differentiation to MFB (Xie et al., 2015). In the prostate, however, TGF-β1-induced fibroblast-to-MFB transdifferentiation led to suppression of pyruvate kinase, PKM2 (Untergasser et al., 2005). In cancer-associated fibroblasts, the glycolytic product lactate is associated with increased stemness, and constitutive TGF-β receptor activation led to metabolic reprogramming with increased lactate production (Martinez-Outschoorn et al., 2011; Guido et al., 2012). This suggests that lactate may be crucial in TGF-β-mediated HSC activation. In line, baboon liver MFB treated with lactate had an increased intracellular proline pool and upregulated collagen synthesis (Savolainen et al., 1984). Since MFB produce high amount of collagen (Bataller and Brenner, 2005), it will be interesting to test whether glucose-derived lactate could modulate human or murine liver MFB, including analysis on the role of lactate dehydrogenases in this context. Together, the currently available information suggests that glycolysis is critical in MFB physiology and offers hints for further investigations.

### Tricarboxylic acid (TCA) cycle

The role of TCA in HSC activation or MFB function is still unclear and the relevance of most TCA enzymes is yet to be delineated. It is also unclear if loss of lipid droplets (LDs), which occurs during HSC activation (Blaner et al., 2009; Kluwe et al., 2011), is aimed at supplying acetyl-CoA for TCA via β-oxidation. The sole evidence of TCA involvement in HSC activation stems from a recent study showing that succinate induces G protein-coupled receptor 91 (GPR91) to increase the production of TGF-β, collagen type I, and α-SMA (Li et al., 2015). This link between succinate and HSC activation suggests a likely relevance of TCA intermediates in metabolism and signaling regulation during HSC-MFB transdifferentiation. Noteworthy, succinate level is significantly increased in lung MFB and fibrotic lungs. Succinate accumulation enhanced TGF-β1-induced HIF-1α stabilization and MFB differentiation (Xie et al., 2015). Thus, it will be interesting to further investigate the effect of other TCA intermediates, including α-ketoglutarate that was recently shown to maintain pluripotency in embryonic stem cells via epigenetic control (Carey et al., 2015). Other interesting intermediates are fumarate and 2-hydroxyglutarate, which are called “oncometabolites” due to their oncogenic effect on rapidly proliferating cells (Xu et al., 2011; Sullivan et al., 2013; Nowicki and Gottlieb, 2015). Whether these “oncometabolites” exert profibrotic effects on MFB is yet to be explored. On a broader perspective, any abnormal accumulation of TCA intermediates may alter transcriptomic or signaling networks to initiate or sustain MFB phenotype.

### Glutamine metabolism

Glutamine is a very abundant amino acid and a highly energy-rich metabolite in humans. Research on mechanisms of cellular glutamine flux have rapidly evolved in recent years to elucidate its relevance in cell metabolism—e.g., in sustaining nucleotide biosynthesis, TCA, and lipogenesis (DeBerardinis et al., 2007; Metallo et al., 2012; Mullen et al., 2012; Son et al., 2013). In HSCs, the mechanism of glutamine utilization or its relevance to activation is currently unknown. Nevertheless, activated HSCs are long known to express high glutamine synthetase (GS; Bode et al., 1998). GS expression and glutamine metabolism have been severally linked to the Wnt/β-catenin signaling pathway (Cadoret et al., 2002; Austinat et al., 2008; Schmidt et al., 2011; Karner et al., 2015). Thus, the evidence that activation of the Wnt pathway elevates GS expression, while suppressing HSC activation marker α-SMA (Kordes et al., 2008) suggests that Wnt may control HSC fate by modulating glutamine metabolism. However, since the authors measured neither the intracellular nor extracellular glutamine level in their experiment, it is hard to discuss the effect of the elevated GS on glutamine metabolism. Therefore, further studies are needed to clarify the role of glutamine in activation and MFB bioenergetics, including any prospects of targeting glutamine utilization in MFB.

### Fatty acid metabolism

Fatty acids (FA) are important in liver physiology, notably in the maintenance of membrane integrity, signaling, energy production, and regulation of inflammation in various cellular and tissue compartments (Freigang et al., 2013; Bazinet and Layé, 2014; Serhan, 2014). When deregulated, FA metabolism accounts for several liver diseases such as hepatic steatosis, steatohepatitis, and cirrhosis (Rinella, 2015). Specifically, polyunsaturated fatty acids (PUFAs) as substrates for the cyclooxygenase pathway vitally regulate initiation and resolution of inflammation (Alhouayek and Muccioli, 2014; Buckley et al., 2014), and have been linked to HSC activation. Rat HSCs at early activation stage replace retinyl esters with PUFAs in LDs (Testerink et al., 2012). However, the mechanism by which the incorporated PUFAs later contribute to HSC activation is not yet known, especially given that HSCs loose LDs during activation (Blaner et al., 2009).

Saturated FA such as oleic acid (OA) and palmitic acids (PA) also participate in MFB activity (Lee et al., 2010, 2014). Further, palmitate and retinol supplementation suppress activation of human immortalized LX-2 (Xu et al., 2005) and primary human HSCs (Lee et al., 2010). Lee et al. found that palmitate and retinol induced adipose differentiation-related protein (ADRP), which regulates the formation of LDs. Mechanistically, ADRP induction led to LDs formation, and the suppression of activation and fibrogenic targets including α-SMA, collagen, and MMP1 (Lee et al., 2010). The effect of OA and PA on increasing lipid storage was also corroborated by a recent study, in which these FA were reported to further synergize with natural compounds, such as rutin and curcumin, to increase LDs and suppress proliferation of HSCs (Lee et al., 2014). On the contrary, OA treatment induced TGF-β, which ostensibly promoted the MFB phenotype in mesangial cells by inducing the expression of collagen I, fibronectin,and α-SMA (Mishra and Simonson, 2008); whether OA similarly induces TGF-β in HSCs is yet unknown. Besides activating MFB, lipids may participate in MFB-mediated inflammatory functions. For instance, human liver MFB were found to trigger activation of monocytes by secreting prostaglandin-E2 (PGE2) *in vitro*. Accordingly, blocking PGE2 production with cyclooxygenase 2 inhibitor (NS-398) reduced the expression of the monocyte marker CD163 (Zhang et al., 2014).

Evidences also suggest that FA regulates MFB activation and function in other tissues. For instance, arachidonic acid and docosahexaenoic acid (DHA) reversed the MFB phenotype of valvular interstitial cells from porcine aortic valves by decreasing contractility and expression of α-SMA via a mechanism involving suppression of RhoA/G-actin/MRTF signaling (Witt et al., 2014). In the prostate, DHA also suppressed fibroblast to MFB differentiation. Specifically DHA prevented TGF-β-induced differentiation, α-SMA expression, and migration of prostate associated fibroblasts (Bianchini et al., 2012). Furthermore, DHA suppressed matrix metalloproteinase 2 (MMP2) release and reversed the myofibroblast phenotype of prostate adenocarcinoma-associated fibroblasts (Bianchini et al., 2012). In other studies, dietary supplementation with fish oil blocked cardiac fibroblast activation and prevented cardiac fibrosis. Accordingly, eicosapentaenoic acid and DHA increased cyclic GMP levels, attenuated cardiac fibroblast transformation, proliferation, and collagen synthesis, and also blunted TGF-β1-induced phospho-Smad2/3 nuclear translocation through activation of cyclic GMP/protein kinase G pathway (Chen et al., 2011). Nitrated fatty acids (NFAs), formed when nitric oxide (NO) and NO-derived species react with unsaturated FA, are critical mediators of signaling and inflammation-related functions (reviewed by Trostchansky and Rubbo, 2008). NFAs upregulated PPARγ and blocked TGF-β signaling/activity in human lung fibroblasts (Reddy et al., 2014). *In vivo*, NFA treatment led to reduction of disease severity and reversal of existing MFB numbers and collagen deposition in a mouse model of pulmonary fibrosis (Reddy et al., 2014). Furthermore, resolvins, a family of lipid mediators derived from omega-3 PUFA, and known to have anti-inflammatory potency (Xu et al., 2010), inhibited interstitial fibrosis by blocking proliferation of resident fibroblasts (Qu et al., 2012). Despite these evidences that fatty acids influence the MFB phenotype, no study has directly interrogated the role of key enzymes in fatty acid metabolism, including FASN, ACLY, ACC in HSC activation. Hence, further studies will help to resolve the exact molecular regulation and relevance of fatty acid metabolism in HSC biology.

### Cholesterol metabolism

Recently, it was shown that cholesterol accumulation drives liver fibrosis (Tomita et al., 2014). According to the authors, increased cholesterol intake in a model of non-alcoholic steatohepatitis (NASH) led to free cholesterol accumulation in HSCs. Cholesterol accumulation consequently sensitized HSCs to TGF-β-induced activation by upregulating toll-like receptor 4 protein (TLR4), which suppressed TGF-β pseudoreceptor Bambi (Tomita et al., 2014). Similarly, in hypercholesterolemic mice with aortic valve disease, rapid normalization of cholesterol levels by genetic switching led to normalized superoxide levels, decreased myofibroblast activation, and a suppressed disease progression (Miller et al., 2009). The molecular mechanism by which cholesterol mediates activation and fibrosis is not fully understood. However, blocking cholesterol metabolism has offered prospects in ameliorating MFB-mediated fibrogenesis (Table 1).

**Table 1:** **Summary of selected research findings on liver myofibroblast metabolism, autophagy. and epigenetics with notes on evidence of therapeutic prospects**.

**Molecular process**	**Findings/Evidence**	**Models**	**^1^Evidence of therapeutic prospects with inhibitor (s)?**	**References**
Glycolysis	•↑Glycolytic phenotype and targets (GLUT1, HK2, PKM2) during HSC differentiation ↑Number of glycolytic stromal cells	1° mouse HSCs (*in vitro*) MCD (*in vivo*) BDL (*in vivo*)	Yes—with 2-deoxy glucose	Chen et al., 2012
	•Hedgehog inhibitors suppress activation and also lactate output and glycolytic targets (e.g., HK, PFK2, and Glut4)	1° rat HSCs (*in vitro)* CCl4-induced rat fibrosis liver (*in vivo)*	No	Lian et al., 2015
TCA/Glutamine metabolism	•Succinate treatment increases α-SMA via GPR91 activation	LX-2 and 1° mouse HSCs cultured in MCD (*in vitro*)	No	Li et al., 2015
	•Stimulation of β-catenin-dependent Wnt signaling prevents HSC activation	1° rat HSCs (*in vitro*)	Yes—with GSK3β inhibitor TWS119	Kordes et al., 2008
	•↑Glutamine synthetase (GS) in activated HSCs GS as potential marker of HSC activation	1° rat HSCs (*in vitro*)	No	Bode et al., 1998
Fatty acid/Cholesterol metabolism	•HSCs replace retinyl esters with PUFAs in lipid droplets during activation process ↑Incorporation of exogenous arachidonic acid	1° rat HSCs (*in vitro*)	No	Testerink et al., 2012
	•Palmitate suppress activation by ↑ADRP	LX-2 and human 1° HSCs (*in vitro*)	No	Lee et al., 2010
	•↑Accumulation of oleic and palmitic acids increases autophagy in activated HSCs	LX-2 (*in vitro*)	No	Lee et al., 2014
	•Simvastatin—↓HSC proliferation, ↓collagen I, revert HSCs to quiescence	1° rat HSCs (*in vitro*)	Yes—with Simvastatin	Rombouts et al., 2003
	•Fluvastatin—↓palmitate-induced HSC activation; alleviated steatosis-induced HSC activation; ↓hepatic fibrogenesis	Rat immortalized HSCs (T6 cells; *in vitro*) NASH (*in vivo*)	Yes—with Fluvastatin	Chong et al., 2015
	•Atorvastatin attenuates HSC activation and fibrosis	BDL mice (*in vivo*)	Yes—with Atorvastatin	Trebicka et al., 2010
	•Inflammatory and profibrotic function and effect of leptin was blocked by inhibition of NADPH oxidase	1° human and mouse HSCs (*in vitro*)	Yes—with NADPH oxidase inhibitor diphenylene-iodonium (DPI)	De Minicis et al., 2008
Autophagy	•Autophagy promotes fibrogenesis Blocking autophagy via Atg7 suppress liver fibrosis	1° mouse and human HSCs Mouse immortalized HSCs (JS1; *in vitro*) *Atg7 transgenic* mice (*in vivo*) CCl4 and TAA-treated mice (*in vivo*)	Yes—with 3-Methyladenine (3-MA)	Hernández-Gea et al., 2012
	•Inhibition of autophagy suppress HSC activation	1° mouse HSCs (*in vitro*)	Yes—with Bafilomycine A1	Thoen et al., 2011
Epigenetics	•JMJD1A—novel epigenetic regulator in HSCs ↓JMJD1A correlates with reinforced H3K9me2 in the PPARγ gene promoter, ↑α-SMA and collagen	CCl4-treated mice (*in vivo*)	No	Jiang et al., 2015
	•Epigenetic silencing of Smad7 enables TGF-β1-induced fibrosis via Smad2/3	Rat HSCs (*in vitro*)	Yes—with 5-aza-2'-deoxycytidine (5-azadC)	Bian et al., 2014
	•Ethanol induce multiple epigenetic regulators, including a histone 3 lysine 4 (H3K4) methyltransferase (MLL1) during HSC activation	1° rat HSCs (*in vitro*)	No	Page et al., 2015
	•VDR ligands inhibit TGF-β1-induced HSC activation by blocking recruitment of histone modifiers (CBP and p300) and histone H3 hyperacetylation in profibrotic genes	^*^LX-2 cells 1° rat and mouse HSCs Vdr^−∕−^ mice (*in vivo*) CCl4-treated mice (*in vivo*)	Yes—with Vit-D agonist Calcipotriol	Ding et al., 2013
	•MRTF-A mediates fibrosis by recruiting histone methyltransferase complex to the promoters of fibrogenic genes to activate transcription	1° mouse HSCs (*in vitro*) HSC-T6 cells (*in vitro*) MRTF-A KO mice treated with CCl4 (*in vivo*)	No	Tian et al., 2015
	•HDAC inhibition blocks activation markers α-SMA, lysyl oxidase, collagens.	1° mouse HSCs (*in vitro*) ^**^CCl4-treated mice (*in vivo*)	Yes—with HDAC class II inhibitor, MC1568	Mannaerts et al., 2013
	•HDAC inhibition blocks HSC proliferation, activation, and suppress liver fibrosis	1° mouse and human HSCs (*in vitro*) BDL rat (*in vivo*)	Yes—with HNHA	Park et al., 2014
	•HDAC inhibition blocks HSC activation and fibrosis	1° mouse HSCs (*in vitro*) CCl4-treated mice (*in vivo*)	Yes—with Valproate	Mannaerts et al., 2010

1 Refers only to part of the study showing inhibitors that directly target a gene or pathway in the respective molecular process; 1°, Primary; BDL, Bile duct ligation; CCl4, Carbon tetrachloride; MCD, Methionine choline-deficient; MRTF, Myocardin-related transcription factor; NASH, Non-alcoholic steatohepatitis; TAA, Thioacetamide ↑, increase or upregulation; KO, knockout; ↓, decrease or downregulation; HNHA, N-hydroxy-7-(2-naphthylthio)heptanomide;

* The main model used to demonstrate the antagonistic role of VDR ligand on TGFβ1-induced activation;

** In vivo data was inconclusive due to variabilities within samples (Mannaerts et al., 2013).

Statins are known blockers of cholesterol metabolism and act by inhibiting 3-hydroxy-3-methyl-glutaryl-CoA reductase (HMGCR). HMGCR catalyzes the rate-limiting step in cholesterol metabolism (i.e., HMG-CoA → mevalonate), which commits acetyl-CoA to cholesterol production (Sirtori, 2014). Compelling evidences suggest that commonly used statins, such as simvastatin, pravastatin, fluvastatins, and atorvastatin are beneficial in targeting fibrogenesis. For instance, simvastatin suppressed HSC activation and liver fibrosis by increasing endothelial nitric oxide synthase expression, while suppressing the expression of inducible nitric oxide synthase (Wang et al., 2013)—a proinflammatory mediator (Brenner et al., 2013). Simvastatin also suppressed rat HSC proliferation and collagen I production, and reversed the morphology of activated HSCs toward quiescence (Rombouts et al., 2003). Further, pravastatin in combination with protein kinase c (PKC) inhibitor (enzastaurin) yielded a synergistic antifibrotic effect in *in vitro* and *in vivo* liver fibrosis models, notably by inducing cell apoptosis (Yang et al., 2010). Fluvastatin was recently shown to suppress palmitate-induced HSC activation *in vitro.* In addition, fluvastatin suppressed inflammation and oxidative stress to ameliorate steatosis-induced HSC activation and hepatic fibrogenesis in *in vivo* NASH model (Chong et al., 2015). Recently, Trebicka et al. (2010) investigated the antifibrotic effect of atorvastatin in rats after bile duct ligation (BDL). In their study, early (prophylactic) therapy with atorvastatin significantly reduced fibrosis and HSC activation. On the contrary, late atorvastatin therapy (against severe fibrosis) transduced HSCs into a more quiescent state, and led to suppression of MMP2 and profibrotic targets (e.g., TGF-β1, CTGF, and PDGFβ-R), but without affecting inflammation and fibrosis (Trebicka et al., 2010). In addition to suppressing activation, atorvastatin also induced senescence in MFB, both *in vitro* and *in vivo* as determined by p21 expression and β-galactosidase staining (Klein et al., 2012). Together, these evidences show that cholesterol-lowering agents have antifibrotic potency. It is noteworthy that the regulatory mechanisms linking statins to MFB deactivation are still vaguely defined. Studies suggest that besides inhibiting cholesterol metabolism, statins may suppress activation by attenuating membrane Ras and cytosolic RhoA levels (Rombouts et al., 2003; Porter et al., 2004). Statins may also mediate their antifibrotic effects by activating the transcription factor Kruppel-like factor 2 (KLF2; Marrone et al., 2013, 2015). In addition, the findings that atorvastatin exerted divergent effects depending on timing of therapy (Trebicka et al., 2010) suggest that the stage of fibrosis may determine the mechanism of action or effects of statins on MFB.

Besides inhibition of HMGCR, reduction of cholesterol levels by other mechanisms may also be of therapeutic benefits in limiting fibrogenesis. For instance, ezetimibe, which inhibits cholesterol absorption, was found to improve hepatic fibrosis in a controlled trial of 80 non-alcoholic fatty liver disease patients (Takeshita et al., 2014). Noteworthy, many targets in carnithine metabolism/transport and steroid biosynthesis, including *CPT1A, CPT1B, SQLE, SREBF, SC5DL*, and *HMGCS1* were deregulated in this patient cohort. However, despite genomic evidence of suppressed HSC to MFB transition, patients treated with ezetimibe had adverse effects, including increased long-chain fatty acid and glycated hemoglobin (HbA1c), which led to premature termination of the study (Takeshita et al., 2014). It remains to be elucidated how the cholesterol biosynthetic pathway modulates MFB features, and no study has yet reported the role of cholesterogenic targets like *HMGCS, HMGCR*, and *FDFT1* in HSC activation and MFB function.

### Oxidative stress and anti-oxidant defense system

Oxidative stress and the cellular anti-oxidant defense system are regulated in a coordinated fashion during inflammation. It is known that reactive oxygen species (ROS) such as hydrogen peroxide and superoxides are released and scavenged during hepatic wound healing (Prosser et al., 2006). This process must be coordinated by a plethora of tightly regulated mechanisms to ensure homeostatic balance. Some well-studied anti-oxidant mediators, such as NADPH Oxidase, galectin-3, glutathione, and superoxide dismutases are involved in MFB physiology and could be prospective targets in fibrotic therapy.

*NADPH Oxidase 4 (NOX4)* is a key mediator of the cellular antioxidant system, which is known to be upregulated in fibrosis and linked to TGF-β fibrotic action. Targeted inhibition of NOX4 suppresses HSC activation and also the initiation or progression of fibrogenesis in other organs including lung, breast, kidney, and heart (Cucoranu et al., 2005; Aoyama et al., 2012; Sancho et al., 2012; Chan et al., 2013; Hecker et al., 2014; Manickam et al., 2014; Sampson et al., 2014; Tobar et al., 2014; Lan et al., 2015). In line, pharmacological and genetic inhibition of NADPH oxidase in HSCs blocked inflammatory and profibrotic functions of leptin, e.g., enhanced HSC proliferation; up-regulation of fibrogenic markers, inflammatory mediators, and chemokine expression, thus supporting a role of NOX in mediating fibrogenesis via signaling control (De Minicis et al., 2008).

*Galectin-3* is a pleiotropic β-galactoside-binding lectin. Galectin-3 associates with cell adhesion molecules to mediate its downstream functions such as cell apoptosis, adhesion, migration, angiogenesis, fibrosis, and inflammatory responses (Li et al., 2014). Galectin-3 is overexpressed upon injury and regulates hepatic progenitor cell expansion and HSC activation (Henderson et al., 2006; Hsieh et al., 2015). In addition, several studies show that galectin-3 expression has direct correlation with HSC phagocytosis, matrix production, and hepatic fibrosis (Maeda et al., 2003; Henderson et al., 2006; Jiang et al., 2012; Martínez-Martínez et al., 2014). Recently, galectin-3 was found to function as a scavenging receptor for advanced lipoxidation endproducts (ALEs) in the liver. Consequently, galectin-3 deficient mice fed with atherogenic diet present with less steatosis and reduced tissue uptake of ALEs (Iacobini et al., 2011).

*Glutathione* is another prominent player in the cellular antioxidant function (Lu, 2013; Espinosa-Diez et al., 2015). Increased glutathione suppresses HSC growth and activation (Fu et al., 2008). Accordingly, stimulation with TGF-β suppressed expression of glutamate-cysteine ligase (GCL), the rate-limiting enzyme in glutathione biosynthesis, leading to lower glutathione levels in cultured HSCs (Fu et al., 2008). This suggests that suppression of glutathione levels is a mechanism of TGF-β-induced fibrosis. Similarly, increased intracellular glutathione levels in lung fibroblast inhibit Smad3 phosphorylation to suppress TGF-β1-induced profibrotic effects, such as expression of CTGF, collagen I, fibronectin, and transformation to MFB (Ono et al., 2009).

*Superoxide dismutase (SOD)* is also suggested to have antifibrotic activity. A study with a skin fibrosis model indicates that exogenous Cu/Zn SOD exerts antifibrotic activity by suppressing MFB features, such as expression of α-SMA, TGF-β1, and ECM (Vozenin-Brotons et al., 2001). Manganese superoxide dismutase (MnSOD) is a downstream target of the AKT-dependent forkhead transcription factor FOXO1 (Adachi et al., 2007). MnSOD induction via active FOXO1 partly inhibits HSC proliferation and transdifferentiation by suppressing ROS production (Adachi et al., 2007), further showing that anti-oxidant mediators are crucial in HSC activation and MFB function.

In human alcoholic hepatitis, disturbance of the antioxidant system occurs in the background of advanced fibrosis (Colmenero et al., 2007). Those patients present with significant accumulation of fibrogenic MFB and overexpression of genes involved in oxidative stress, including NOX4, as well as dual oxidases 1 and 2. Oxidative stress is also associated with corneal and alveolar MFB functions (Yang et al., 2013b; Vyas-Read et al., 2014). Furthermore, production of mitochondrial complex III ROS is essential for TGF-β-driven MFB differentiation and profibrotic gene expression in human lung fibroblasts (Jain et al., 2013). However, ROS scavengers trigger TGF-β1-mediated differentiation of human subcutaneous fibroblasts into MFB (Cat et al., 2006; Popova et al., 2010). Taken together, oxidative stress and anti-oxidant mediators are pivotal in activation and MFB function. Therefore, metabolic processes that generate or remove ROS, e.g., oxidative phosphorylation, pentose pathways, and glutathione metabolism, may critically participate in liver MFB activities and so represent yet untapped areas in the search for antifibrotic therapies.

## Autophagy—a prospective facet in liver myofibroblasts pathophysiology

Autophagy, literarily meaning “self-eating,” is a rapidly emerging facet in cellular bioenergetics. It defines a process whereby cells eat up their cytoplasmic components in order to generate metabolites for energy sustainability (Green and Levine, 2014; Hurley and Schulman, 2014). In normal and disease states, autophagy has critical survival, protective, and immune modulatory functions—the latter including suppression of proinflammatory cytokines (Levine et al., 2011; Choi et al., 2013). There are several known markers of autophagy, including the most studied ATG8/LC3, and SQSTM1/p62, ATG1/ULK1, ATG9, and BECN1/ATG6 (Klionsky et al., 2012). However, little and conflicting information currently exist on the role of autophagy in HSCs, MFB, and fibrosis as discussed below.

### Autophagy is profibrotic

Recent findings implicate autophagy as promoter of liver fibrosis (Mallat et al., 2014; Lee et al., 2015). Increased expression of autophagy markers positively correlates with ductular reaction (Hung et al., 2015), a process that goes hand in hand with HSC activation in a subset of liver diseases, and thus may directly participate in tissue repair and hepatic fibrogenesis (Williams et al., 2014). Further, autophagy markers, notably microtubule-associated protein 1 light chain 3B (LC3B), ATG12-5, and ATG7 were significantly upregulated in the livers of cirrhotic patients and in 2-acetylaminofluorene (AAF)/CCl_4_-induced liver fibrosis in rat (Hung et al., 2015). With LC3B as marker, the authors showed that autophagy correlated with severity of fibrosis and was consistently increased in cirrhosis regardless of varying etiologies. However, while the autophagy markers correlated with protein expression of α-SMA and bile duct proliferation marker CK19, a direct overlap between MFB and autophagy was not apparent in this study, as immunofluorescent staining showed no co-localization of LC3B with α-SMA in α-SMA+ MFB (Hung et al., 2015). Considering that lipids are among its metabolic triggers (Galluzzi et al., 2014), autophagy may induce HSC activation by crosstalk with lipid molecules. Indeed, autophagy facilitates loss of LDs and concomitantly promotes the supply of free fatty acids as energy-building substrates during HSC activation (Hernández-Gea et al., 2012). Consequently, inhibition of autophagy with 3-Methyladenine (3-MA) decreased ATP levels in HSCs. Furthermore, blocking autophagy by interfering with its Atg7 attenuated CCl_4_ or thioacetamide-induced liver fibrosis and matrix accumulation (Hernández-Gea et al., 2012). The reduction of ECM accumulation and fibrosis upon loss of autophagic function in mouse HSCs led to the suggestion to target autophagy in fibrotic diseases (Hernández-Gea et al., 2012). Consistent with this, earlier studies had shown that inhibition of autophagy in cultured hepatocytes and in mouse liver led to increased triglyceride storage and LDs (Singh et al., 2009). Furthermore, autophagy was significantly increased upon HSC activation, while treatment with autophagy inhibitor Bafilomycine A1 blunted HSC activation (Thoen et al., 2011). Treatment of rat AAF/CCl_4_ fibrotic model with chloroquine, which blocks autophagic degradation in the lysosome, also ameliorated liver injury, decreased the expression of CK19 and pro-fibrogenic targets (COL1A1, α-SMA, TGF-β), and blunted liver fibrosis (Hung et al., 2015). These findings suggest that autophagy is relevant in MFB generation and is potentially druggable toward inhibition of excessive MFB function.

### Autophagy is antifibrotic

Contrary to a direct correlation between autophagy and HSC activation, autophagy has also been found to be protective in fibrosis (Mallat et al., 2014). For instance, mutations in the autophagy gene, *Atg5*, apparently interfered with HSC-to-MFB transdifferentiation to protect mice against chronic CCl4-induced liver fibrosis (Lodder et al., 2015). Atg5^−∕−^ mice treated with CCl4 had higher hepatic levels of interleukins (IL-1A, IL-1B), enhanced inflammatory cell recruitment, and were more susceptible to liver fibrosis (Lodder et al., 2015). Indeed, in studying the potential therapeutic benefits of natural compounds in alleviating fibrosis, it was observed that activated HSCs have increased light chain I/II (LC3 I/II) protein expression when pre-treated with fatty acids (OA and PA) and then post-treated with various natural compounds, including rutin and curcumin (Lee et al., 2014). While the study confirms that FA induces autophagy as mentioned earlier, the subsequent conclusion that the natural compounds are potential antifibrotic agents (Lee et al., 2014) seems to suggest that autophagy induction (as caused by the compounds) is antifibrotic in activated HSCs. Similarly, tonsil-derived mesenchymal stem cells could ameliorate CCl4-induced liver fibrosis in mice via autophagy activation, notably by reducing TGF-β and type I collagen expression (Park et al., 2015). In pulmonary fibrosis, reduced autophagy in aged animals also worsened the fibrotic phenotype (Sosulski et al., 2015). In addition, TGF-β promotes lung fibrosis by suppressing autophagy (Sosulski et al., 2015). Taken together, autophagy may represent a highly context-dependent facet in MFB pathophysiology. Whether autophagy is protective or induces cell death may largely depend on the initiating factor. Supporting this view, Rautou and colleagues argued that in most liver diseases, autophagy is mainly protective, e.g., by allowing the degradation of LDs in fatty acid disease and protein aggregates in alcohol liver disease. Contrarily, Hepatitis B/C virus can subvert autophagy for their replicative advantage (Rautou et al., 2010). Here, it is worthy to highlight that the loss of LDs attributed to “protective” autophagy (Rautou et al., 2010) is also a mechanism through which autophagy provides energy substrates to promote HSC activation and fibrosis (Hernández-Gea et al., 2012). Therefore, it will be of interest to further interrogate protective and detrimental autophagy in HSC activation, MFB functions, and in the switch between activation and dedifferentiation to quiescence (Figure 1).

## Epigenetic alterations in liver myofibroblasts

Epigenetics refer to heritable traits resulting from chromosomal alterations that do not alter DNA sequence. Epigenetic alterations maintain cell identity and include DNA methylation, histone modifications, chromatin remodeling, transcriptional control, and post-translational modification of non-coding RNA (Berger et al., 2009; Cedar and Bergman, 2009; Portela and Esteller, 2010; Mann, 2014). Interestingly, several epigenetic targets, including DNA methyltransferases (DNMTs), histone deacetylases (HDACs), histone methyltransferases (*DOT1L, EZH2, G9A*), histone demethylases (JmjC-domain proteins, *LSD1*), and binding domains (*BET, BAZ2B, L3MBTL1*) are druggable in human diseases (Helin and Dhanak, 2013). Epigenetic alterations occur in liver fibrosis and chronic liver diseases (Mann, 2014; Atta, 2015; Lleo et al., 2015) and are relevant in HSC activation (Kang et al., 2015).

### DNA methylation

During activation, HSCs accumulate methylation changes that significantly modulate the expression of genes involved in cell activation and inflammation (Götze et al., 2015). Specifically, expression of DNA methyltransferases, DNMT3A and DNMT3B, increased with HSC activation (Götze et al., 2015). One consequence of hypermethylation is gene silencing. For instance, transcriptional silencing of PPARγ, which occurs during HSC activation (Hazra et al., 2004), has been attributed to methylation based epigenetic control (Mann et al., 2007; Yang et al., 2012). Recently, the Jumonji Domain-Containing Protein 1A (JMJD1A)—a histone H3K9 demethylase—was found to regulate HSC activation and liver fibrosis by targeting PPARγ gene expression (Jiang et al., 2015). Knockdown of JMJD1A in HSCs correlated with reinforced H3K9me2 in the PPARγ gene promoter; increased α-SMA and collagen expression, and enhanced necrosis in the CCl_4_ mouse fibrosis model (Jiang et al., 2015). Consistent with this finding, blocking CpG methylation with the nucleotide analog 5-aza-2′-deoxycytidine (5-azadC) prevented loss of PPARγ expression (Mann et al., 2007). Methylation-based control in HSCs is also evident from methyl-CpG-binding protein (MECP2), known to repress chromatin structures. MECP2 is induced during HSC activation, correlates with α-SMA expression and contributes to MFB transdifferentiation by regulating fibrogenic targets (Mann et al., 2007; Yang et al., 2013a). Mechanistically, MECP2 repressed Patched (PTCH1), whose loss upon hypermethylation is necessary for sustained fibroblast activation and liver fibrosis (Yang et al., 2013a). Furthermore, DNA methylation is responsible for epigenetic silencing of Smad7, which enables fibrogenic TGF-β effects via Smad2 and Smad3 phosphorylation (Bian et al., 2014). Hence, RNA interference and 5-azadC-mediated inhibition of the methylation gene *DNMT1* prevented TGF-β-induced proliferation and upregulation of activation markers in HSCs (Bian et al., 2014). More studies are required to further validate methylation switches at various MFB differentiation stages under normal and perturbed microenvironments.

### Histone modification

Histone modification is another active epigenetic alteration during HSC activation. For instance, HSCs in a mouse model of acute liver failure secrete IL-1, which induces high MMP9 levels, leading to collagen IV degradation (Yan et al., 2008). If uncontrolled, MMP9 expression could oppose MFB-mediated accumulation of ECM. Hence maintenance of appropriate balance is necessary during HSC activation or MFB function. Interestingly, epigenetic repression of MMPs has been suggested as a mechanism that controls HSC transdifferentiation (Qin and Han, 2010). Consequently, MMP9 and MMP13 promoters in MFB display impaired histone acetylation and assembly of transcription machinery. These alterations blocked docking of transcription factor c-Jun on the MMP promoters (Qin and Han, 2010). Similarly, ectopic expression of *HDAC4* in quiescent HSCs suppressed intrinsic and IL-1-induced MMP promoter activity and repressed MMP9 expression. These findings implicate accumulation of HDACs at MMP promoters, specifically *HDAC4*, as an epigenetic mechanism to repress MMP expression during HSC activation (Qin and Han, 2010). Similar regulation is provided via *HDAC7*, which represses hepatocyte growth factor (HGF) and thus increases susceptibility to hepatocellular damage, inflammation, and fibrosis in liver injury (Pannem et al., 2014). *HDAC7*-mediated repression of HGF in HSCs is antagonized by the tumor suppressor gene cylindromatosis (*CYLD*). Accordingly, *CYLD* interacts with and removes HDAC7 from the HGF promoter, hence enabling HGF induction, which subsequently is secreted and protects against hepatocellular injury and fibrosis (Pannem et al., 2014). In cultured human skin fibroblasts, *HDAC6, HDAC8*, but most potently *HDAC4* were identified as crucial epigenetic regulators of TGF-β-induced MFB differentiation, ostensibly by blocking the expression of TGF-β signaling repressors 5′-TG-3′-Interacting Factor (TGIF) and TGIF2 (Glenisson et al., 2007).

Recently, the myocardin-related transcription factor (MRTF), ethanol and vitamin D receptor (VDR) have been identified as epigenomic modifiers during HSC activation. MRTF promotes MFB differentiation, fibrosis, and TGF-β-induced HSC activation (Crider et al., 2011; O'Connor and Gomez, 2013; Velasquez et al., 2013; O'Connor et al., 2015; Sisson et al., 2015). Mechanistically, MRTF-A mediates fibrosis via recruitment of the histone methyltransferase complex to the promoters of fibrogenic genes and subsequent transcriptional activation (Tian et al., 2015). Ethanol exposure was found to promote rat HSC transdifferentiation by inducing global changes in histone modifying enzymes that upregulate ECM components elastin (*ELN*) and collagens (Page et al., 2015). The authors found that ethanol induced the expression of histone 3 lysine 4 (H3K4) methyltransferases, mainly *MLL1*. *MLL1* binding was enriched on *ELN* gene promoter and consequently induced *ELN* expression in transitioning HSCs. In addition, *MLL1* expression also correlated with *ELN* and collagens in ALD liver explants further confirming that ethanol induced pro-fibrogenic processes via epigenetic regulators (Page et al., 2015). VDR ligands also induce chromatin remodeling as a mechanism to counteract TGF-β-driven HSC activation (Ding et al., 2013). TGF-β induced activation by promoting the recruitment of histone-modifying cofactors, p300 and CBP, and by promoting histone H3 hyperacetylation at a VDR/SMAD co-occupied regulatory region of *COL1A1*. Treatment with VDR ligands antagonized activation by disrupting TGF-β-mediated SMAD/VDR interaction. Consequently, synthetic VDR agonist Calcipotriol reduced collagen deposition and fibrotic gene expression *in vitro* and *in vivo* (Ding et al., 2013). These studies underscore the critical role of histone modification in HSC transdifferentiation (Figure 1).

Indeed, evolving links between epigenetics and MFB function justifies targeting histone modifiers in antifibrotic therapies. In line, various HDAC inhibitors are effective against TGF-β-induced MFB generation (Glenisson et al., 2007; Guo et al., 2009; Liu et al., 2013), including MC1568, valproic acid (VPA), trichostatin A (TSA), and butyrate. MC1568 inhibited HSC activation markers, such as type I/III collagen, SMA, and lysyl oxidase. In addition, MC1568 induced antifibrotic microRNA-29 and also suppressed the proliferation of freshly isolated mouse HSCs (Mannaerts et al., 2013). Unfortunately, the authors were unable to reproduce the result in CCl_4_ fibrosis model owing to technical issues, probably due to inefficient delivery or fast metabolization of the drug (Mannaerts et al., 2013). VPA also suppresses liver fibrosis and HSC activation *in vitro* and *in vivo* (Mannaerts et al., 2010; Aher et al., 2015). However, given the pivotal role of TGF-β in HSC activation, it is important to mention that VPA did not interfere with early TGF-β targets, SMAD 6 and 7 (Mannaerts et al., 2010), thus raising further questions on the exact mechanism, by which epigenetics influences HSC activation by TGF-β. Furthermore, TSA and RNA interference against *HDAC4* prevented MFB differentiation as measured by α-SMA expression (Glenisson et al., 2007). Nilotinib, a tyrosine kinase inhibitor, selectively induces apoptotic and autophagic cell death in HSCs by blocking HDAC 1, 2, and 4 (Shaker et al., 2013). Other HDAC inhibitors that suppress HSC activation include a chalcone derivative 2',4',6'-tris(methoxymethoxy) chalcone (TMMC; (Lee et al., 2011)), and N-hydroxy-7-(2-naphthylthio)heptanomide (HNHA; (Park et al., 2014)). HNHA not only suppressed HSC proliferation, activation and liver fibrosis, but also restored liver function and prolonged survival in the BDL rat model (Park et al., 2014). Further evidence for targeting HDACs in fibrotic diseases have been shown in other settings, e.g., cardiac and lung fibroblasts (Zhang et al., 2013; Sanders et al., 2014; Schuetze et al., 2014). Together, these data highlight the relevance of epigenetics in HSC activation and encourage the exploitation of epigenetic targets in the control of MFB-mediated fibrogenesis.

## Technical advances that could help delineate metabolism, autophagy, and epigenetics in liver myofibroblasts

Within the last few decades, several cutting edge techniques have emerged for the study of complex biological processes.

In metabolism, mass spectrometry based metabolomics techniques have been developed for measuring metabolic flux. Consequently, it is now possible to precisely determine utility of metabolites with a very high degree of precision (Zamboni et al., 2009; Hiller and Metallo, 2013). Thus, metabolic flux analyses could enable (a) holistic and simultaneous quantification of labeled and unlabeled metabolites derived from parent carbon sources (e.g., glucose or glutamine), (b) help to delineate the metabolic properties of MFB at different differentiation stages, and (c) offer hints on prospective metabolic pathways of therapeutic relevance in HSC activation or MFB function. In addition, cellular respiration can be easily measured by fiber optic oxygen sensors, clark electrode, and extracellular flux analyzers—the latter offering the advantage of assessing oxygen consumption and extracellular acidification rates in living cells (Zhang et al., 2012; Perry et al., 2013). Furthermore, *in vivo* measurement of metabolism using hyperpolarized, (13)C-labeled cells has been successfully applied (Rodrigues et al., 2014; Brindle, 2015). The latter *in vivo* approach could enhance accuracy given the challenges of potential artifacts from cell cultures.

For autophagy studies, techniques such as time-lapse microscopy (Muzzey and van Oudenaarden, 2009) and transmission electron microscopy could be adopted with molecular biology methods (Klionsky et al., 2012) to dissect how autophagy affects the evolution of fibrosis. This could help identify novel potential mediators of MFB autophagic mechanisms for therapeutic purposes. Also, research on MFB autophagy will benefit from other advances in microscopy, e.g., super-resolution microscopy that allow high-resolution imaging and protein tracking in living cells (Bergner et al., 2013; Barden et al., 2015; Chéreau et al., 2015). Tools for measuring autophagy based on biochemical features, e.g., quenching of GFP fluorescent signals in the lysosome at low pH (Bampton et al., 2005), could enable a more improved understanding of autophagy during HSC transdifferention (Figure 1).

Delineating epigenetic alterations in HSCs will be greatly enhanced by bisulfite conversion, chromatin immunoprecipitation and high throughput DNA methylome analyses (Shull et al., 2015; Tang et al., 2015). Other techniques, including single cell analysis, sequencing techniques, and multi-color fluorescence activated cell sorting could be applied to uncover yet unknown epigenetic alterations specific to HSC activation and MFB functions (Figure 1).

To accelerate understanding of MFB function, it is important to consider and possibly tackle the fact that *in vivo* changes in gene expression during HSC activation may differ markedly from those that occur in *in vitro* culture (De Minicis et al., 2007). Such differences may be due to a plethora of factors, including but not limited to cell culture conditions, contamination by other liver cell populations, and sample handling. Where possible, a strategy to overcome this challenge would be to interface the assays mentioned above with lineage tracing. Lineage tracing has been successfully applied as a powerful innovative tool for tracking MFB origin (Mederacke et al., 2013; Lua et al., 2014; Swiderska-Syn et al., 2014). Hence, lineage tracing opens up the feasibility of *in vivo* studies of alterations in HSCs at quiescent, transitory, and activated states, and will highly complement genomic and functional assays.

Finally, optimal application of novel gene editing techniques, such as CRISPR/Cas9, TALENs, etc. (Cho et al., 2014; Jamal et al., 2015; LaFountaine et al., 2015; Laufer and Singh, 2015) will hugely accelerates the identification and understanding of metabolic, autophagic, and epigenetic targets in MFB, especially when complemented with proteomic and transcriptomic profilings. Ultimately, perhaps the most important technical step in understanding MFB physiology, especially in the context of metabolism, autophagy, and epigenetics, is to explore all possible strategies to eliminate analytical variables that distort results.

## Uncharted territories in the study of metabolism, autophagy and epigenetics in liver myofibroblasts

Currently, few studies have focused on how MFB feed, regulate survival via autophagy or via epigenetic alterations that activate or silence key genes in spatio-temporal cell fate decisions. Consequently, knowledge of metabolism, autophagy, and epigenetics in MFB is still at a very nascent stage with many convoluted parts worth further clarifications (Figure 1). The studies that so far focused on the above subjects have offered exciting platforms for further questions. However, more efforts should be dedicated to delineate their molecular relevance to MFB origin and function in health and disease. Lessons can also be learned from other settings, e.g., cancer. For example, metabolism has evolved as a potentially druggable process in cancer entities and many metabolic targets are in preclinical and clinical trials (Galluzzi et al., 2013). Whether those therapeutic strategies would find application in MFB origin/liver fibrosis remains an open question. It is also unknown which metabolic priorities are exploited by MFB, e.g., particular substrates that are indispensable for their survival. The timing of metabolic alterations is also critical to any studies, as changes in mRNA transcripts occur within hours in cultured HSCs (Chen et al., 2012). Unresolved questions also abound in the area of nutrient exchange between MFB and other liver cell populations in the microenvironment, including the extent of their capacity to sustain *de novo* anabolism. Regarding autophagy, scientific efforts should clarify boundaries between “self-eating” autophagy to provide energy building substrates and autophagy with a self-destructive consequence. Questions like “how autophagy markers contribute to MFB status” remain hugely unanswered. Similar questions in metabolism and autophagy also apply to epigenetics. Many epigenomic targets are druggable and several epigenomic drugs yield a beneficial response in MFB and fibrosis. Hence, understanding how DNA methylation and histone modification control HSC transdifferentiation could substantially improve the prospects of better therapeutic interventions. Furthermore, questions also arise on possible crosstalks or feedback loops or overarching control by potential regulators that are chiefly responsible for MFB physiology. These may include transcription factors, microRNAs, long non-coding RNAs, and other regulators of the genome. Hence, it is yet unclear under which circumstances certain regulators switch on/off metabolism, autophagy, or epigenetic modifiers in MFB (Figure 1). Ultimately, there are currently no established metabolic, autophagy, or epigenetics markers in MFB. Dissecting these uncharted territories will substantially open new windows for therapeutic interventions in MFB-mediated fibrosis.

## Conclusion

In the light of evolving molecular insights, metabolism, autophagy, and epigenetics are critical players in HSC activation and MFB functions. Currently available data lead us to propose that transcriptional and epigenetic controls likely coordinate metabolism and autophagy in HSC to MFB transdifferentiation (Figure 1). In the context of the discussed molecular processes, more studies are required to deepen understanding of MFB origin and function in liver fibrogenesis. We suggest that ongoing and future MFB research should interrogate the relevance of key metabolic enzymes, autophagy markers, and epigenetic modifiers, including but not limited to those mentioned here and already investigated in cancer (Claus and Lübbert, 2003; Cheong et al., 2012; Popovic and Licht, 2012; Galluzzi et al., 2013; Helin and Dhanak, 2013; Table 1). We also recommend that researchers should critically consider the time points selected for MFB studies since unforeseen switch from activation to quiescence or vice versa could obscure molecular details. In the end, a broad-spectrum integration of cutting edge tools that enable simultaneous measurements, such as “omics” technologies, will enable better understanding of MFB and further expose novel regulators or biomarkers of MFB activity. We conclude that a detailed understanding of metabolism, autophagy and epigenetics in liver MFB will inspire a new frontier in the development of antifibrotic therapy.

## Author contributions

ZCN, HA, and YL conceived and wrote the manuscript, while SW, SD, and YL provided comments, revised, and corrected the manuscript.

## Funding

SD is supported by funds from the Deutsche Forschungsgemeinschaft (DFG) Do373/13-1, the BMBF programs “Virtual Liver” (Grants 0315755, 0315764), and LiSyM (Grant PTJ-FKZ: 031L0043), as well as from Marie Curie Actions of the European Union's Seventh Framework Programme (FP7/2007-2013) Grant PITN-GA-2012-316549 (IT LIVER: Inhibiting TGF-beta in liver diseases). SW is supported by BMBF SysTox Grant (FKZ 031A303E). ZCN is a recipient of Ph.D. Scholarship from the Niger Delta Development Commission, Nigeria, and appreciates the generous supports from HBIGS, University of Heidelberg, Germany.

### Conflict of interest statement

The authors declare that the research was conducted in the absence of any commercial or financial relationships that could be construed as a potential conflict of interest.

## Abbreviations

3PO: Cell-permeable inhibitor of PFKFB3
ACC: Acyl CoA carboxylase
ACLY: ATP citrate lyase
ALD: Alcoholic liver disease
ADRP: Adipose differentiation-related protein
BDL: Bile duct ligation
CCl4: Carbon tetrachloride
CTGF: Connective tissue growth factor
DHA: Docosahexaenoic acid
ECM: Extracellular matrix
FA: Fatty acid
FASN: Fatty acid synthase complex
FDFT1: Farnesyl-diphosphate farnesyltransferase 1
GLUT1: Glucose transporter 1
GSK3β: Glycogen synthase kinase 3 beta
H3K9me2: Histone H3 dimethyl Lys9
HCC: Hepatocellular carcinoma
HDAC: Histone deacetylase
HK2: Hexokinase 2 (muscle isoform)
HMGCR: 3-hydroxy-3-methylglutaryl-Coenzyme A reductase
HMGCS: 3-hydroxy-3-methylglutaryl-Coenzyme A synthase 1
HSCs: Hepatic stellate cells
IL: Interleukin
LDs: Lipid droplets
LX-2: immortalized human hepatic stellate cells
MCT4: Monocarboxylate transporter 4
MFB: Myofibroblasts
MMP: Matrix metalloproteinase
NASH: Non-alcoholic steatohepatitis
OA: Oleic acid
PA: Palmitic acid
PPARγ: Peroxisome proliferator-activated receptor gamma
PFKFB3: 6-phosphofructo-2-kinase/fructose-2,6-biphosphatase 3
PKM2: Pyruvate kinase M2 (muscle isoform)
PUFAs: Polyunsaturated fatty acids
ROS: Reactive oxygen species
TCA: Tricarboxylic acid
TGF-β: Transforming growth factor beta
VPA: Valproic acid
α-SMA: alpha smooth muscle actin.
